# Immortalized Mesenchymal Stromal Cells Overexpressing Alpha-1 Antitrypsin Protect Acinar Cells from Apoptotic and Ferroptotic Cell Death

**DOI:** 10.21203/rs.3.rs-2961444/v1

**Published:** 2023-08-09

**Authors:** Sara Shoeibi, Erica Green, Hua Wei, Wenyu Gou, Charlie Strange, Hongjun Wang

**Affiliations:** MUSC; MUSC; MUSC; Medical University of South Carolina; MUSC; MUSC

## Abstract

Chronic pancreatitis (CP) is a progressive inflammatory disorder that impairs endocrine and exocrine function. Our previous work suggests that mesenchymal stem/stromal cells (MSCs) and MSCs overexpressing alpha-1 antitrypsin (AAT-MSCs) could be therapeutic tools for CP treatment in mouse models. However, primary MSCs have a predisposition to undergo senescence during culture expansion which limits their therapeutic applications. Here we generated and characterized immortalized human MSCs (iMSCs) and AAT-MSCs (iAAT-MSCs) and tested their protective effect on 2,4,6-Trinitrobenzenesulfonic acid (TNBS) -induced acinar cell death in an in vitro cell culture system. Primary MSCs were immortalized by transduction with simian virus 40 large T antigen (SV40LT), and the resulting iMSC and iAAT-MSC lines were analyzed for proliferation, senescence, phenotype, and multi-differentiation potential. Subsequently, the impact of these cells on TNBS-induced cell death was measured and compared. Both apoptosis and ferroptosis pathways were investigated by assessing changes of critical factors before and after cell treatment. Coculture of iMSCs and iAAT-MSCs with acinar cell lines inhibited early apoptosis induced by TNBS, reduced ER stress, and reversed TNBS-induced protein reduction at tight junctions. Additionally, iMSCs and iAAT-MSCs exerted such protection by regulating mitochondrial respiration, ATP content, and ROS production in TNBS-induced acinar cells. Furthermore, iMSCs and iAAT-MSCs ameliorated ferroptosis by regulating the ferritin heavy chain 1 (FTH1)/protein disulfide isomerase (PDI)/glutathione peroxide 4 (GPX4) signaling pathways and by modulating ROS function and iron generation in acinar cells. These findings identified ferroptosis as one of the mechanisms that leads to TNBS-induced cell death and offer mechanistic insights relevant to using stem cell therapy for the treatment of CP.

## INTRODUCTION

Chronic pancreatitis (CP) involves inflammatory cell recruitment, activation of pancreatic stellate cells, and subsequent acinar cell apoptosis, and necrosis and fibrosis [1, 2]. Because pancreatic acinar cells constitute the largest population of parenchymal cells in the pancreas, the accumulation of excess amino acids in these cells in response to hormonal stimulation may cause pancreatic toxicity and contribute to the onset and progression of CP by mechanisms that are not yet fully understood [5, 6]. Alterations in cellular pathways in the acinar cells, a major cell type of exocrine pancreas, offer insights into the molecular events that occur during the early stages of CP [6, 7]. Identifying the underlying cellular mechanisms involved in acinar cell death in CP is crucial for understanding its pathophysiology and developing novel therapeutic options. Apoptosis is known to play a crucial role in CP, destroying acinar cells and other cells in the pancreas, which in turn leads to the disruption of exocrine function. Oxidative stress is also implicated in the pathogenesis of CP, as increased generation of superoxide anions (O^2−^) and reactive oxygen species (ROS) are chronically activated in CP [8, 9]. The main effectors of apoptosis, such as caspases and the B cell lymphoma 2 (Bcl-2) family, may regulate the progression of CP by linking the outer and inner pathways that promote apoptosis [9, 10]. While many studies have examined the role of apoptosis in various pancreatitis models, the specific cell death pathways and their relationship to acinar cell loss in CP remain unclear [11–14].

Ferroptosis is a regulated form of cell death associated with lipid peroxidation and disrupted redox homeostasis [10–12]. The process of ferroptosis is triggered by intracellular iron overload and the inactivation of glutathione peroxide 4 (GPX4) [10, 13]. Although ferroptosis has been investigated in the development of various diseases, such as ischemia-reperfusion injury [15], ulcerative colitis [16], and colorectal cancer [20], its role in the development of chronic pancreatitis has not been reported. A recent study demonstrated that the downregulation of GPX4 by the transcription factor AP-1 is critical in the aggravation of acinar cell ferroptosis during the progression of acute pancreatitis [21]. Whehter ferroptosis is involved in acinar cell death in CP has yet to be studied. Therefore, we hypothesis that ferropotis plays a critical role in TNBS-induced acinar cell death and the inhibition of ferroptosis may be a potential therapeutic option for treating CP and other diseases associated with ferroptosis.

Mesenchymal stromal cells (MSCs) have therapeutic potential due to their immunoregulatory and anti-inflammatory effects [15–18]. Overexpressing alpha-1 antitrypsin (AAT) in MSCs shows promise in anti-inflammatory properties. [16, 19–21]. However, like other adult stem cells, MSCs undergo telomere shortening with each cell division. Various types of cellular stress can lead to chromosomal instability, DNA damage accumulation, and the acquisition of a senescent phenotype [26]. Several studies have shown that culture-expanded MSCs have decreased proliferation, lower expression of specific cell surface markers, limited differentiation potential, and are prone to senescence *in vitro*, thus compromising their therapeutic usefulness [26–28]. To overcome *in vitro* senescence of aged MSCs, lines of immortalized MSCs (iMSCs) have been created by transducing immortalizing genes such as simian virus 40 large T antigen (SV40LT) and human papillomavirus E6/E7 genes, which have attracted significant research interest [26, 29]. It is believed that genetic alterations that promote cell cycle progression and suppress stress-induced senescence are required for MSC immortalization [27, 29]. Immortalizing MSCs with immortalizing genes is a promising approach to overcoming senescence [22–24].

This study generated immortalized MSC lines (iMSCs and iAAT-MSCs) and investigated their effects on apoptosis and ferroptosis induced by TNBS in acinar cell lines. iMSCs and iAAT-MSCs inhibited TNBS-induced ferroptosis by suppressing endoplasmic reticulum (ER) stress and ROS production. These immortalized MSCs hold promise for CP cell therapy.

## MATERIAL AND METHODS

### Cell Culture

Rat pancreatic AR42J acinar cells (pancreatoma, ATCC CRL-1492) were cultured in F-12K medium (ATCC, USA) supplemented with 20% fetal bovine serum (FBS; Thermo Fisher Scientific, MA, USA) and 1% penicillin/streptomycin (Gibco, MT, USA) at 37°C with 5% CO_2_ until they reached over 80% confluency. Cells were detached using 0.25% trypsin solution containing 2.2 mM EDTA (Gibco, MT, USA). Human bone marrow-derived MSCs were derived from bone marrow specimen purchased from Stemexpress (Folsom, CA, USA). The donor was a 25-year-old healthy African American male. MSCs were separated from bone marrow as described previously [25], Cells were cultured in low glucose DMEM (Gibco, MT, USA) supplemented with 10% FBS and 1% penicillin/streptomycin at an initial density of 5000 cells/cm^2^.

We generated human AAT-engineered MSCs (hAAT-MSCs) by transducing hMSCs with the pHAGE-CMV-a1aT-UBC-GFP-W lentiviral vectors as previously described [20]. MSCs transduced with the control virus, pHAGE-UBC-GFP-W, were used as MSC control.

### Immortalization of Primary MSCs and AAT-MSCs

We generated immortalized cell lines of MSCs and AAT-MSCs using SV40 T antigen (ALSTEM, CA, USA) at passages 4–5. The target cells were transduced with 20 µl/well of SV40LT viral supernatant in the presence of 4 µl TransPlus reagent and selected using puromycin (Gibco, MT, USA). Two weeks after selection, clones were chosen for expansion and screening. MSCs and AAT-MSCs at P8 or P9 and iMSCs and iAAT-MSCs at P18-P20 were used in this study.

### Coculture of iMSC and iAAT-MSC with AR42J cell lines

AR42J cells were seeded in the bottom well of the Transwell 6-well plates (Corning, NY, USA) at a density of 0.3×10^6^ cells/cm^2^. iMSCs or iAAT-MSCs at a density of 0.6×10^6^ cells/cm^2^ were added on the insert of Transwells in a complete medium for 24 hours. The medium was then changed to DMEM supplemented with 5% FBS and TNBS at 0.10%, or 0.15% for controls (TNBS only) and two treatment groups (TNBS + iMSCs or iAAT-MSCs). Cells cultured alone without TNBS were used as healthy cell controls. After 24 hours of coculture, AR42J cells were collected for further analysis.

### Microscopic Analysis

#### Senescence-Associated β-Galactosidase Activity

Senescence assay was performed on iMSCs and iAAT-MSCs (passage 18), primary MSCs, and AAT-MSCs (passage 9) using the Senescence β-Galactosidase Staining kit (Cell Signaling Technology, MA, USA). Cells were incubated in β-Gal staining solution (final concentration 1 mg/mL, pH 6.0) overnight. The percentage of senescent cells was determined by counting ß-Gal-positive and total cells in five randomly selected microscope fields.

#### Colony-Forming Unit-Fibroblast Assay (CFU-F)

In the colony formation assay, MSCs were plated at densities of 50, 100, and 200 cells per 35 mm well in triplicate. After two weeks of incubation with regular medium replacements, cells were washed, fixed with ice-cold 100% methanol (Thermo Fisher Scientific, MA, USA), and stained with 0.5% crystal violet (Sigma-Aldrich, MO, USA). CFU-F colonies larger than 3 mm in diameter were counted under a light microscope.

#### Preservation of Multipotency in iMSCs and iAAT-MSCs

MSCs, AAT-MSCs (P9), iMSCs, and iAAT-MSCs (P20) were subjected to chondrogenic, osteogenic, and adipogenic differentiation assays using specific kits from Gibco (MT, USA). Differentiation was induced by culturing the cells in specific differentiation media for 14 days (adipogenic) or 22 days (osteogenic and chondrogenic), with regular medium replacements. Subsequently, staining methods were employed to assess differentiation outcomes: 0.5% Oil Red-O solution (Sigma-Aldrich, MO, USA) for adipogenesis, 2% Alizarin red S (pH 4.2, Sciencell, CA, USA) for osteogenesis, and 1% Alcian blue (Lifeline Cell Technology, CA, USA) for chondrogenesis following protocols recommended by the manufacturers.

#### Detection of Intracellular Iron

Prussian blue staining was performed to evaluate intracellular iron content. AR42J cells were cultured with or without iMSCs/iAAT-MSCs for 24 hours. Subsequently, the cells were treated with 0.15% TNBS for an additional 24 hours. After washing with distilled water, the cells were stained with iron solution according to the manufacturer's instructions (Iron Stain Kit; Abcam, MA, USA). A nuclear fast red solution was used for counterstaining. Using a light microscope, the percentage of iron-positive cells was determined by examining five randomly selected fields of view per sample.

#### Immunocytochemistry

To assess the expression of SV40LT and GPX4, cells were fixed with 4% paraformaldehyde, permeabilized with 0.3% Triton X-100, and blocked with 1% bovine serum albumin (Thermo Fisher Scientific, MA, USA). Immunostaining was performed using specific primary and secondary antibodies listed in Table 1. Finally, samples were mounted, and fluorescence micrographs were captured using a Confocal Leica TCS SP5 X microscope (Leica-microsystem, Wetzlar, Germany).

MSC surface marker expression in iMSCs and iAAT-MSCs by flow cytometry

For flow cytometric analysis, iMSCs and iAAT-MSCs were detached, washed, and incubated with PE-labeled antibodies against CD29, CD90, CD44, and CD45 (Table 1) in an FACS buffer (Thermo Fisher Scientific, MA, USA). After washing, cells were analyzed using a Cytoflex flow cytometer (Beckman Coulter, IN, USA), and FlowJo software (BD Biosciences) was used for data analysis. The results are presented as a percentage of positive cells compared to the non-stained controls.

#### Cell apoptosis assay by flow cytometry

Cell apoptosis was assessed using an Annexin V FITC Apoptosis Detection kit (BioLegend, CA, USA). Cells were harvested, washed, and mixed with FITC-conjugated APC Annexin V (2.5 µl) and Propidium Iodide Solution (5 µl) and incubated for 20 mins in the dark at room temperature. The stained cells were then analyzed with flow cytometry.

#### Detection of ROS Production

To detect ROS production, the intensity of green fluorescence emitted by converting rhodamine 123 upon reaction with ROS was measured as previously described [26]. In brief, after treatment with TNBS, AR42J cells were incubated with 5 µM Dihydrorhodamine (DHR)-123 (Sigma-Aldrich, MO, USA). Cells were collected, and ROS production was quantified using flow cytometry. Fluorescence was measured at 485/528 nm on a Bio-Tek (VT, USA) spectrophotometer after 20 min of incubation and every 20 min after the first measurement to identify the maximal production of reactive nitrogen oxide species (RNOS)/ROS.

#### Measurements of mitochondrial respiratory activity

After treatment with TNBS, AR42J cells were transferred to an XF96 culture plate at a density of 8×10³ cells per well. Oxygen consumption rate (OCR) and extracellular acidification rate (ECAR) were measured using the Seahorse XF analyzer (Agilent Technologies, CA, USA). The XFp Cell Mito Stress A test kit was used for OCR measurements, involving different cycles of injections of oligomycin, FCCP, and rotenone/antimycin A. ECAR was measured using a program that included glucose, oligomycin, and 2-deoxy-D-glucose injections [24].

#### Glutathione Peroxidase Activity (GPX) Assay

GPx activity was analyzed using a colorimetric assay kit (Abcam, MA, USA) following the manufacturer's protocol. Briefly, AR42J cells were collected after treatment, and incubated with glutathione reductase (GR) and reduced glutathione (GSH). GPx activity was assessed by adding cumene hydroperoxide and measuring the absorbance at 340 nm.

#### Lipid Peroxidation Assay

The relative concentration of malondialdehyde (MDA) was determined in AR42J cell lysates using the Lipid Peroxidation Assay Kit (Abcam, MA, USA). The assay involved reacting MDA with thiobarbituric acid, which was quantified at 352 nm using a microplate reader.

### Molecular Analysis

Approximately 1.2×10^6^ cells were used to extract total cellular RNA with an RNA extraction kit (Qiagen, MD, USA). A 1 µg aliquot of the extracted RNA was used for a reverse transcription-polymerase chain reaction (RT-PCR) with the iScript cDNA Synthesis Kit (Bio-Rad, CA, USA). Previously described human primers for cell differentiation were used [27] (see Table 2). Quantitative real-time polymerase chain reaction (qPCR) was performed in triplicate with specific primers for the genes listed in Table 2 using a CFX-96 Real-Time PCR system thermal cycler and SYBR green Mastermix (Bio-Rad, CA, USA). The expression levels of glyceraldehyde-3-phosphate dehydrogenase (GAPDH) were used for normalization. qPCR data were analyzed using LightCycler 96 Relative Quantification software (Bio-Rad).

### Western blot analysis

Cultured cells were washed with PBS and lysed in protein lysis buffer containing protease and phosphatase inhibitors (Sigma-Aldrich, MO, USA). The supernatant was collected, and the total protein concentration was measured using a BCA Protein Assay kit (Thermo Scientific, MA, USA). Protein samples were separated by SDS–PAGE using 10% polyacrylamide gels and transferred onto PVDF membranes. The membranes were blocked and incubated with primary antibodies overnight, followed by incubation with horseradish peroxidase-conjugated secondary antibodies (Table 1). Protein signals were imaged using a ChemiDocTM Imaging System (Bio-Rad, CA, USA) and quantified with ImageJ software (NIH). Ponceau S Solution (Abcam, MA, USA) was used for visualizing total protein on PVDF membranes.

### Statistical analysis

Data are presented as mean ± standard error of the mean (SEM) and analyzed using GraphPad Prism software (Version 9). The Student’s t-test compared the means of the two groups, while ANOVA followed by a post-hoc test compared means belonging to multiple groups. Statistical significance was defined as P < 0.05, and further categorized as P < 0.0001, P < 0.001, and P < 0.01.

## RESULTS

### iMSCs and iAAT-MSCs express SV40LT and common mesenchymal stem cell surface markers

**Figure 1:**
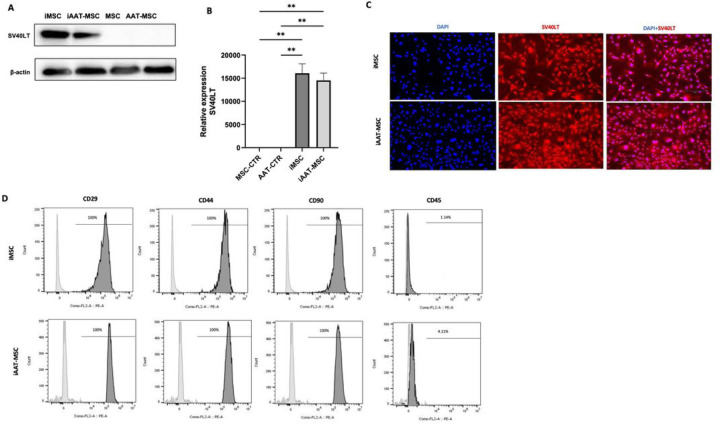
SV40LT and cell-surface marker expression in iMSCs and iAAT-MSCs. SV40LT expression was assessed by (A) western blot and (B) RNA expression compared to primary MSC and AAT-MSC. (C) SV40LT immunostaining of iMSC and iAAT-MSC; SV40LT is shown in red, DAPI staining is shown in blue, Bar=100 µm. **(D)** Surface profile analysis of the cells (P8) using flow cytometry. Representative FACS analysis of iMSC- and iAAT-MSC-defining surface positive marker panel (CD29, CD44, CD90) and CD45 as a surface negative marker. Grey histograms represent unstained controls and the black overlays represent each antigen; percentages of positive cells are shown within histograms. Data represent mean ± SEM of n = 4; The P-values were calculated using one-way ANOVA; **P < 0.01. CTR: control.

We first measured expression of SV40LT in control and iMSCs. SC40LT protein and gene expression was detected in transduced iMSCs and iAAT-MSCs, but not in control MSCs or AAT-MSCs (Fig. 1A,B). Immunostaining confirmed successful transduction with SV40LT, as indicated by red fluorescence specifically observed in the cell nucleoli (Fig. 1C). Flow cytometric results demonstrated high expression of mesenchymal cell markers CD90, CD44, and CD29 (> 95%), and low expression of hematopoietic stem cell marker CD45 (< 4.1%) in the immortalized cell lines (Fig. 1D). These data indicate the successful establishment of iMSCs and iAAT-MSCs, which maintain the characteristic gene expression of mesenchymal stem cell markers.

### iMSCs and iAAT-MSCs retain MSC morphology and show a low percentage of senescent cells

**Figure 2:**
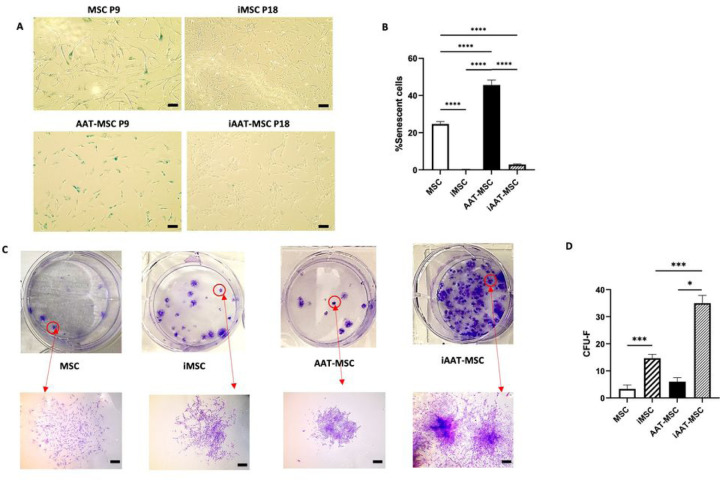
SA-β-gal and crystal violet staining to assess senescence and clonogenic potential of iMSCs and iAAT-MSCs. **(A)** SA-ß-Gal stained iMSC, iAAT-MSC, and MSCs and AAT-MSCs by phase-contrast microscopy. SA-ß-Gal activity is shown in blue, Bar=100 µm. **(B)** MSC morphology of iMSCs, iAAT-MSCs, MSCs, and AAT-MSCs and percentage of senescent cells (ß-Gal staining) at Passage 18. **(C)** Representative plates and total CFU-F in iMSC, iAAT-MSC, MSCs, and AAT-MSCs stained with crystal violet, Bar=200 µm. (D) Numbers of colonies at 14 days after culture in all groups of cells. The error bars represent mean ± SD for each cell line (n = 3); The P-values were calculated using one-way ANOVA;*P < 0.05, ***P < 0.001, ****P < 0.0001.

Late-passage MSCs have been observed to become a heterogeneous population with a high percentage of senescent cells [28]. iMSCs and iAAT-MSCs showed low senescence-associated activity even at passages 20 (Fig. 2A, B). Specifically, 0.19 ± 0.5% of the cells in iMSC and 3.75 ± 1.5% in iAAT-MSCs were ß-Gal-positive. In contrast, in primary MSCs and AAT-MSCs at P9, 24.69% and 45.63% of cells were positive for SA-ß-Gal activity, respectively (Fig. 2B). iMSCs and iAAT-MSCs showed similar β-galactosidase activity (Fig. 2A).

CFU-F assays showed iMSCs and iAAT-MSCs maintained their morphology like MSCs (Fig. 2C). The frequency of CFU-Fs differed significantly between iMSCs and MSCs (P < 0.001) and between iAAT-MSCs and AAT-MSCs (P < 0.05, Fig. 2D). Significantly, iAAT-MSCs exhibited a more robust clonal self-renewal ability compared to iMSCs (P < 0.001).

### iMSCs and iAAT-MSCs maintain capacity for multilineage differentiation

**Figure 3:**
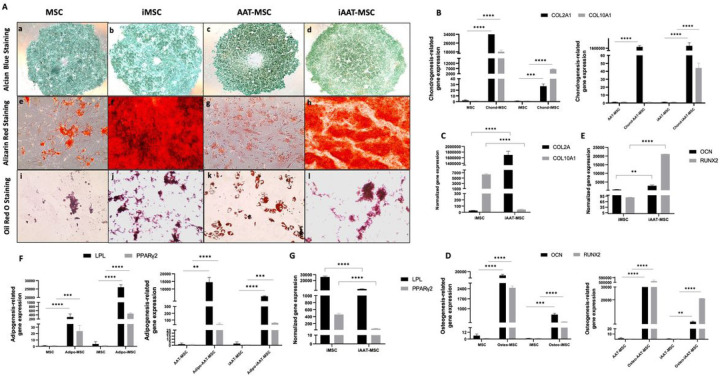
Multilineage differentiation of iMSCs and iAAT-MSCs. **(A)** Chondrogenic differentiation indicated by alcian blue staining for cartilage proteoglycans immunostaining (scale bar = 200 μm); osteogenic differentiation indicated by alizarin red staining for calcium deposit and AP stain for alkaline phosphatase (scale bar = 100 μm); adipogenic differentiation indicated by oil red staining for lipoid deposits (scale bar = 50 μm). **(B)** Chondrogenic differentiation potential of iMSCs, and AAT-MSCs shown by upregulation of chondrogenic markers (Col2A1 and Col10A1) assessed after three weeks of chondrogenesis under pellet culture. **(C)** Chondrogenesis-related gene expression was shown by comparing iMSC and iAAT-MSC. **(D)** osteogenic markers (OCN and RUNX2) were upregulated after three weeks of osteogenesis. **(E)** Osteogenesis-related gene expression was shown by comparing iMSC and iAAT-MSC. **(F)** Adipogenic markers (LPL and PPARγ) were upregulated after two weeks of adipogenesis. **(G)** Adipogenesis-related gene expression was shown by comparing iMSC and iAAT-MSC. GAPDH was used as an internal control. Bars represent mean ± SD; The P-values were calculated using Student’s paired t-test; **P < 0.01, ***P < 0.001, ****P < 0.0001. data represent three independent experiments.

iMSCs and iAAT-MSCs and their non-transduced counterparts (MSCs and AAT-MSCs) retained multipotency (Fig. 3A). Histological staining confirmed the presence of proteoglycans in chondrogenic differentiation (Fig. 3A, a–d), calcium phosphate deposits in osteogenic differentiation (Fig. 3A, e–h), and intracellular lipid droplets (Fig. 3A, i–l) in adipogenic differentiation.

The expression of chondrogenic genes (COL2A1 and COL10A1), osteogenic genes (OCN and Runx2), and adipogenesis-related genes (LPL and PPARγ2) was analyzed in transduced cells and their non-transduced counterparts (Fig. 3B–G). Chondrocyte spheroids of all cell types showed upregulated expression of COL2A1 and COL10A1 compared to undifferentiated cells (Fig. 3B). iAAT-MSCs exhibited higher expression of COL2A1 and lower expression of COL10A1 compared to iMSCs (P < 0.0001, Fig. 3C). However, expression of COL10A1 was higher in iAAT-MSCs chondrocytes compared to AAT-MSCs (P < 0.0001, Fig. 3B). Osteoblast cultures of all cell types showed significant expression of OCN and Runx2 (Fig. 3D), with higher expression in iAAT-MSCs compared to iMSCs (P < 0.01 and P < 0.0001, respectively, Fig. 3E). Adipocytes derived from iMSCs showed higher expression of LPL and PPARγ2 compared to MSCs and iAAT-MSCs (P < 0.0001, Fig. 3F,G). Overall, iMSCs and iAAT-MSCs maintained multilineage potential comparable to MSCs and AAT-MSCs.

### iMSCs and iAAT-MSCs inhibit early apoptosis induced by TNBS in acinar cells

**Figure 4:**
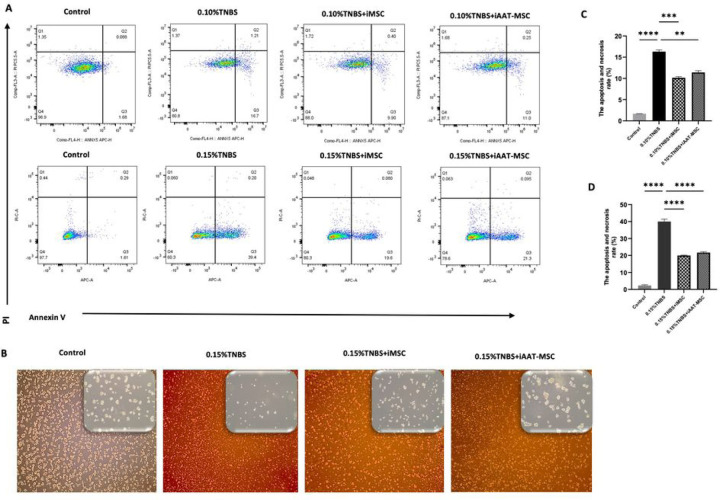
iMSC and iAAT-MSC inhibited cell death of TNBS-damaged acinar cells. (A) AR42J cells were co-cultured with iMSCs or iAAT-MSCs for 24h, AR42J cells were treated with 0.10%, and 0.15% TNBS for another 24 h. AR42J cells were harvested, and then induction of apoptosis was detected using Annexin V-FITC/PI staining assay and flow cytometry. (B) TNBS-damaged AR42J cells (0.15% TNBS) and co-cultured-AR42J cells (with iMSCs or iAAT-MSCs) were incubated in the conditioned medium with 5% FBS for 24 h, then observed using an inverted microscope (Bar= 100 μm and 50 μm). (C) Quantifying the number of apoptotic cells after treatment with 0.10%, and (D) 0.15% TNBS. Data represented the mean ± SEM of three independent experiments (**P < 0.01, ***P < 0.001, ****P < 0.0001 vs. TNBS-damaged AR42J cells).

Treatment with TNBS induced a dose-dependent early apoptosis in AR42J cells (Fig. 4A). However, coculture with iMSCs or iAAT-MSCs significantly decreased early apoptosis rates compared to TNBS treatment alone. When AR42J cells were cocultured with iMSCs or iAAT-MSCs, they exhibited colony formation instead of the single-cell morphology observed in TNBS induction alone (Fig. 4B). Coculturing AR42J cells with iMSCs and iAAT-MSCs showed significant improvement in reducing cell death compared to 0.10% TNBS induction (P < 0.01 and P < 0.001, respectively) (Fig. 4C). The most significant effect was observed when AR42J cells were cocultured with iMSCs and iAAT-MSCs compared to 0.15% TNBS induction alone (both P < 0.0001) (Fig. 4D). Based on these promising results, further experiments were conducted using a TNBS concentration of 0.15%.

#### iMSCs and iAAT-MSCs alleviate ER stress and restore the reduced expression of tight junction proteins induced by TNBS

**Figure 5:**
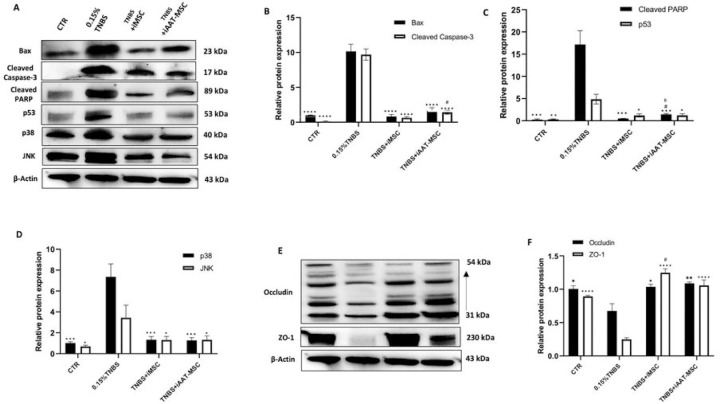
iMSC and iAAT-MSC reduced the apoptosis markers induced by TNBS in acinar cells. **(A)** The expression of cleaved-caspase-3, -PARP, p53, p38, and JNK was evaluated by Western blot and compared among the control, TNBS, TNBS+iMSCs, and TNBS+iAAT-MSCs groups. β-actin was used as the loading control. **(B-D)** The expression of apoptotic proteins was quantified by determining the intensities of the bands compared with those of β-actin. **(E)** Expression of Occludin and ZO-1 proteins in 4 groups of AR42J cells was detected by western blot analysis. β-Actin was used as the loading control. **(F)** Densitometric quantification of tight junction proteins. Data are presented as the mean ± SD. *P < 0.05, **P < 0.01, ***P < 0.001, ****P < 0.0001 vs. TNBS group; #P < 0.05 vs. CTR group; SP < 0.05 vs. TNBS+iMSC group.

**Figure 6:**
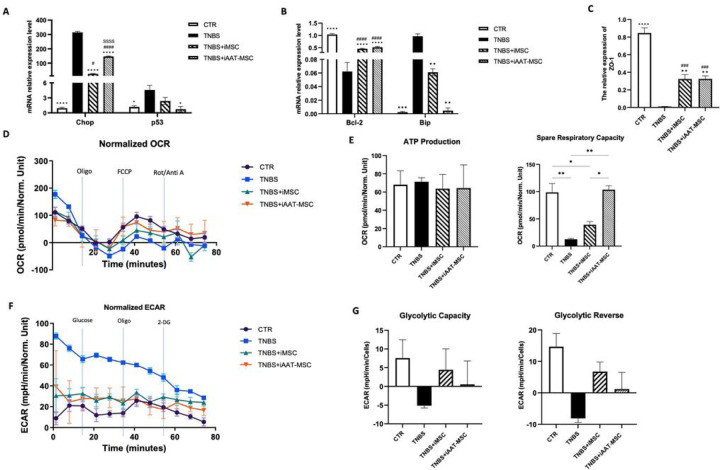
iMSC and iAAT-MSC modulate oxidative stress and regulate mitochondrial respiration and glycolysis. **(A-C)** iMSC and iAAT-MSC modulate oxidative stress and regulate mitochondrial respiration and glycolysis. (A-C) Total mRNA from TNBS treated-AR42J cells in all groups was analyzed by qRT-PCR for the major molecules in the apoptosis signaling pathway: **(A-B)** Chop, p53, Bcl-2, Bip; and **(C)** ZO-1. Expression is presented as a ratio of cytokine/GAPDH. Data are presented as the mean ± SD. *P < 0.05, **P < 0.01, ***P < 0.001, ****P < 0.0001 vs. TNBS group; #P < 0.05, ##P < 0.01, ###P < 0.001, ####P < 0.0001 vs. CTR group; SP < 0.05, SSSP < 0.001, SSSSP < 0.0001 vs. iMSC group. **(D-G)** After 24h, AR42J cells were co-cultured with iMSCs or iAAT-MSCs, treated with TNBS at 0.15% concentration for another 24h, and the dynamics of OCR and ECAR were measured. **(D)** Graphical representation of the OCR measurement over time; A respiratory function stress test was carried out using sequential additions of oligomycin (Oligo, 5 µM), FCCP (5 µM), and rotenone/antimycin A combined (Rot/AA, 2.5 µM) injected seque€ally. **(E)** The effects of co-culture with iMSCs and iAAT-MSCs on the ATP-linked OCR and spare respiratory capacity-linked OCR were calculated from the OCR curves. Data presented as the mean ± standard deviation. *P<0.05; **P<0.01. **(F)** Graphical representation of the ECAR measurement over time; A glycolytic function stress test was carried out using sequential additions of Glucose (10 mM), oligomycin (Oligo, 5 µM), and 2-Deoxy-D-glucose (2-DG, 50 mM). **(G)** The effects of co-culture with iMSCs and iAAT-MSCs on the glycolytic capacity-linked ECAR and glycolytic reserve-linked ECAR calculated from the ECAR curves.

Several reports have suggested that treatment with TNBS induce ER stress [29, 30]. We found here that the exposure to TNBS led to upregulation of pro-apoptotic proteins including caspase-3, Bax, PARP, p53, p38, and JNK in AR42J cells (Fig. 5A–D). Coculturing with iMSCs and iAAT-MSCs significantly suppressed the expression of Bax and cleaved caspase-3 (P < 0.0001, Fig. 5B). Cocultures also resulted in decreased expression of cleaved PARP, JNK, p53, and p38 induced by TNBS treatment (P < 0.05 to P < 0.001) (Fig. 5C,D). In addition, TNBS treatment led to alterations in mRNA levels of CHOP, p53, BiP, and Bcl-2 in AR42J cells. Coculturing with iMSCs or iAAT-MSCs resulted in lower expression of CHOP and BiP and increased expression of Bcl-2 (P < 0.0001) (Fig. 6A,B). Furthermore, treatment with TNBS leads to the disappearance of tight junction proteins zonula occludens-1 (ZO-1) and reduced expression of occludin. In contrast, iMSCs and iAAT-MSCs significantly preserved the expression of ZO-1 and occludin (Fig. 5E,F, 6C). These data suggest that iMSCs and iAAT-MSCs play a crucial role in reducing apoptosis at least in part by preserving tight junction integrity.

### iMSCs and iAAT-MSCs regulate mitochondrial respiration, ATP content in TNBS-treated acinar cells

TNBS-treated acinar cells exhibited mitochondrial damage, leading to a significant reduction in ATP content, indicating impaired mitochondrial function [24] (Fig. 6D,E). The spare respiratory capacity was significantly lower in TNBS-induced AR42J cells compared to the control (P < 0.01, Fig. 6E). Furthermore, the iAAT-MSCs co-cultured acinar cells (but not iMSCs) exhibited a significant better spare respirotry capacity compared to the TNBS only group (P < 0.01, Fig. 6E). TNBS group also showed a high extracellular acidification rate based on ECAR analysis (Fig. 6F). Additional, treatment with iMSCs preserved the glycolytic capacity of acinar cells after TNBS treatment (Fig. 6G), suggesting iMSCs and iAAT-MSCs preserve the mitochondria function of acinar cells.

### iMSCs and iAAT-MSCs regulate mitochondrial respiration, ATP content in TNBS-treated acinar cells

**Figure 7:**
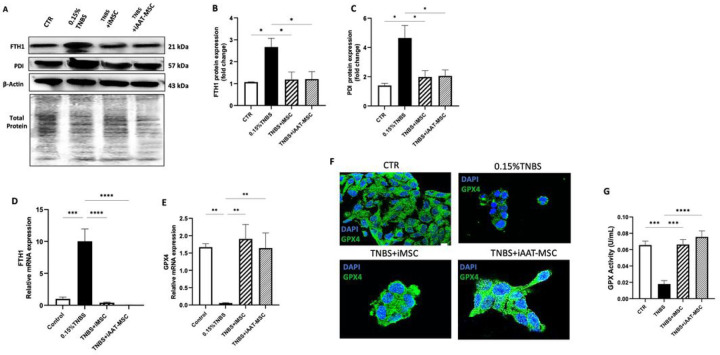
Changes in ferroptosis-related parameters in acinar cells after 24h of TNBS treatment. **(A)** Protein expression levels of FTH1 and PDI in TNBS-induced ferroptosis in AR42J cells detected by Western blotting. **(B-C)**Relative protein expression quantified by densitometry. β-Actin was used as the internal control. **(D-E)**. Total mRNA expression levels of FTH1 and GPX4 in three different groups of TNBS-treated AR42J cells analyzed by qRT-PCR. GAPDH was used as an internal control to normalize the data. **(F)** The total expression level of GPX4 in three different groups of TNBS-treated AR42J cells was monitored by immunofluorescence staining. Green fluorescence intensity reflects the protein expression level of GPX4 (Bar= 25 μm). **(G)**Analysis of glutathione peroxidase (GPx) activity in control (CTR) and three different groups of TNBS-treated AR42J cells. Data are presented as the mean ± standard deviation. **P<0.01, ***P < 0.001, and ****P < 0.0001.

The process of ferroptosis involves primary metabolic processes such as lipid peroxidation and iron generation [32]. We found that TNBS treatment induced ferroptosis in AR42J cells, evident by increased ferritin heavy chain (FTH1) and protein disulfide isomerase (PDI) mRNA and protein levels. However, coculturing acinar cells with iMSCs and iAAT-MSCs effectively suppressed TNBS-induced FTH1 and PD1 expression (Fig. 7A–C), suggesting iMSCs and iAAT-MSCs suppressed TNBS-induced ferroptosis in acinar cells.

Next we measured the expression of GPX4, an antioxidant defense enzyme that is functional to repair oxidative damage to lipids and a leading inhibitor for ferroptosis [39]. GPX4 levels were increased in CP-induced AR42J cells of TNBS plus iMSCs /iAAT-MSCs groups compared to the TNBS group (Fig. 7D,E). Coculturing AR42J cells with iMSCs and iAAT-MSCs preserved GPX activity to a level similar to vehicle-treated control cells (P < 0.001, Fig. 7G). Immunofluorescence staining further confirmed the results (Fig. 7F), suggesting iMSCs or iAAT-MSCs protect acinar cells from ferroptosis via preserving GPX4 expression and activity.

#### iMSCs and iAAT-MSCs suppress TNBS-induced ferroptosis via modulating ROS function and iron generation in acinar cells

**Figure 8:**
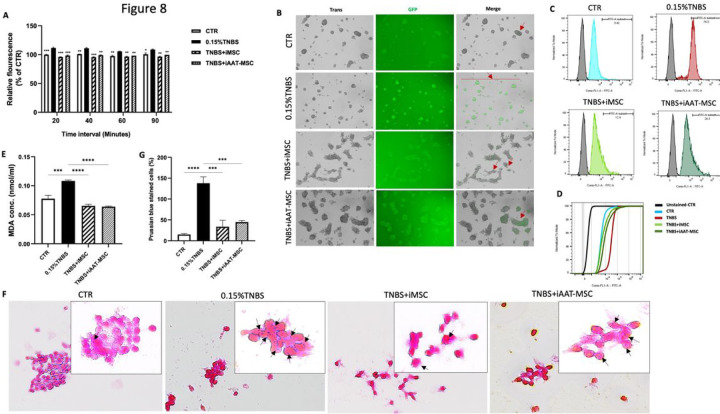
Both iMSC and iAAT-MSC suppressed TNBS-induced ferroptosis via modulating ROS signaling pathway in acinar cells. **(A)** ROS formation was measured by the relationship between the incubation time in the presence of dihydrorhodamine (DHR)-123 and the fluorescence at 485/528 nm after treatment. **(B)**Snapshots from a live cell imaging of four groups of AR42J cells treated with DHR-123. Green fluorescence staining represents ROS production (arrows) once the probe was oxidized to rhodamine-123 (rhod 123) (Bar= 50 μm). **(C)**Flow cytometric analysis of ROS formation on live-gated cells after treatment of AR42J cells in four groups with non-fluorescent DHR-123. **(D)** CDF curve diagram showed the comparable fluorescence signals in four treatment groups of AR42J cells with DHR-123. **(E-G)** Accumulation of lipid peroxidases (MDA) and iron in four groups of AR42€ells. **(E)** MDA levels were determined by MDA Assay Kit. **(F)** Prussian blue assessment of ferrous ions accumulation in treated cells (arrows) (Bar= 50 μm and 25 μm). **(G)** Quantification of iron-positive cells from Prussian blue-stained samples. Data represent the mean ± SEM of at least three independent experiments (*P < 0.05, **P < 0.01, ***P < 0.001, ****P < 0.0001 vs. TNBS-treated AR42J cell group).

To further confirm the capacity of preserving mitochondrial function by iMSCs and iAAT-MSCs, we assessed ROS levels in all groups, as mitochondrial metabolism may affect ROS levels, and mitochondrial DNA damage could increase ROS generation [24, 31]. AR42J cells treated with TNBS had significantly increased cellular ROS levels compared to control cells at different treatment times (P < 0.0001 after 20 min, P < 0.001 after 60 min, and P < 0.05 after 90 min; Fig. 8A). However, cocultures with iMSCs and iAAT-MSCs showed significantly lower fluorescence emission compared to the TNBS group (Fig. 8A). Fluorescence microscopy revealed increased green signal in TNBS-exposed AR42J cells, indicating enhanced conversion of DHR to Rhodamine (Fig. 8B). In addition, TNBS plus iMSCs and TNBS plus iAAT-MSCs treatments reduced the fluorescence intensity of rhodamine 123 by 12.4% and 20.3%, respectively, in comparison to 76.5% in the TNBS-induced group alone (Fig. 8C,D). These findings suggest that coculture of acinar cells with iMSCs and iAAT-MSCs can prevent the burst of intracellular ROS caused by TNBS in acinar cells.

To confirm that ferroptosis was involved in TNBS-induced acinar cells, we measured MDA, an end-product of lipid peroxides and iron, two indicators of ferroptosis [11]. Treatment with TNBS induced a significant increase of MDA content when compared to controls (P < 0.001, Fig. 8E). In contrast, the MDA content was markedly reduced in iMSCs and iAAT-MSCs cocultured groups compared to TNBS group (both P < 0.0001; Fig. 8E). Furthermore, the content of iron in TNBS group increased significantly compared to non-treated controls (P < 0.0001 vs control cells (Fig. 8G), confirming that iron is a crucial part of intracellular lipid peroxidation in ferroptosis caused by TNBS [32]. Coculturing with iMSCs and iAAT-MSCs showed lower iron accumulation in cell cytoplasm (Fig. 8F), and there was a significant difference between treatment groups and non-treatment cells related to iron content (both treatment cells, P < 0.001; Fig. 8G). Our findings of increased lipid peroxidation, ROS production, and iron accumulation following TNBS treatment, along with their suppression by coculturing with iMSCs and iAAT-MSCs, provide evidence supporting the involvement of ferroptosis in acinar cell dysfunction and the mechsnistic insights of iMSCs and iAAT-MSCs.

## DISCUSSION

The short functional lifespan of primary MSCs significantly limits their potentials for use in basic research and in clinical applications, as *in vitro* cultures of MSCs into higher passages can lead to cell senescence. Immortalization of MSCs is a strategy used to overcome their limited lifespan, allowing for unlimited proliferation potential [33–35]. In this study, iMSCs at passge 20 exhibited higher adipogenic potential, while iAAT-MSCs showed greater osteogenic potential. Both cell types displayed comparable chondrogenic differentiation capacity. Similar findings have been reported in previous studies [23, 36–38], indicating that immortalization may alter the differentiation potential of MSCs but still maintain their essential characteristics. Another major findings of this study is that we identified that treatment with TNBS induces ferroptosis in acinar cells and iMSCs and iAAT-MSCs are passage 20 protects acinar cells from ferroptosis.

Previous studies have demonstrated the beneficial effects of MSCs and AAT-MSCs in mitigating pancreatic injury, reducing acinar cell death, and inhibiting CP progression [17, 18, 39, 40]. Due to the strong correlation between ER stress and apoptosis [9], our findings indicated a significant elevation of both ER stress and cell death in the TNBS-induced group. Treatment with iMSCs and iAAT-MSCs effectively protected against these increases. P53, a key controller of cell apoptosis [41], increases the expression of Bax, a proapoptotic Bcl-2 family member, leading to caspase-3 activation during apoptosis [17, 42]. Supplementary Fig. 1 shows the expression of key apoptotic markers, such as Bax, p53, and caspase-3, was elevated in the CP group but decreased after cellular treatment. We also observed a decreased expression of p38 MAPK, PARP, and JNK proteins, along with increased levels of tight junction proteins. Furthermore, the mRNA levels of CHOP and BiP, were significantly reduced in the treated groups. Previous studies have also demonstrated a significant upregulation of expression of apoptosis-related genes during the development of CP [41, 43]. Mitochondrial dysfunction and impaired respiratory complexes were observed in acinar cells exposed to TNBS, leading to decreased cellular OCR and increased ROS levels. This finding suggests that either respiratory complexes are severely damaged or there is a significant metabolic dysfunction in overall mitochondrial biochemistry [13, 24, 44]. However, coculturing with iMSCs and iAAT-MSCs restored mitochondrial activity and ATP production in acinar cells.

Our study revealed that TNBS-induced ER stress contributes to the development of ferroptosis, which is consistent with previous research [45]. Ferroptosis, characterized by an imbalance between oxidation and antioxidant systems [32], involves various molecular factors such as iron levels, ROS, lipid ROS, GPX4, and MDA [11, 13, 45, 46]. A recent study by Wei et al. [10], demonstrated that arsenic induced ferroptosis via the mitochondrial ROS-autophagy pathway, contributing to pancreatic dysfunction. In our investigation, we explored the regulatory mechanisms of ferroptosis and discovered that superoxide free radicals can initiate iron-dependent cell death, a process modulated by the FTH1/PDI/GPX4 system. Wang et al. [32] demonstrated that PDI plays a role in ferroptosis by contributing to the accumulation of lipid ROS. Damage to the GPX repair system leads to lethal accumulation of ROS, while lipid peroxidation promotes the production of ROS [11, 46, 47]. Our study revealed that iMSCs and iAAT-MSCs have the potential to decrease PDI expression and inhibit the accumulation of lipid peroxides. Furthermore, Taha et al [17] showed that BM-MSCs effectively block free radicals induced by L-arginine in pancreatitis treatment. Our findings also support the therapeutic effects of bone marrow-derived iMSCs and iAAT-MSCs in inhibiting the ferroptotic pathway by chelating superoxide free radicals.

Our study not only confirms the fundamental characteristics of bone marrow-derived iMSCs and iAAT-MSCs, including their localization and multilineage differentiation potential, but also demonstrates their therapeutic potential in treating the CP phenotype in a TNBS-induced acinar cell model. By coculturing these immortalized cells with AR42J cells, we found that the activation of regulatory mechanisms of apoptotic and ferroptotic cell death in AR42J cells was inhibited. Thus, our findings provide valuable insights into the pathogenesis and treatment of CP and suggest that iMSCs and iAAT-MSCs may represent a promising target for CP treatment.

## Supplementary Material

Supplement 1
