# Supplementary material for: Immortalized Mesenchymal Stromal Cells Overexpressing Alpha-1 Antitrypsin Protect Acinar Cells from Apoptotic and Ferroptotic Cell Death
Source: Res Sq. 2023 Aug 9:rs.3.rs-2961444. Preprint. [Version 1] doi: 10.21203/rs.3.rs-2961444/v1 (PMC10441457; doi:10.21203/rs.3.rs-2961444/v1)
Supplement: Supplement 1 [file NIHPPrs2961444v1-supplement-1.pdf]

Supplementary Files

This is a list of supplementary files associated with this preprint. Click to download.

- Supp.Fig.1.jpg
- Table1and2.docx
